# Supplementary material for: Outcomes of COVID-19 Hospitalized Patients Previously Treated with Renin-Angiotensin System Inhibitors
Source: J Clin Med. 2020 Oct 28;9(11):3472. doi: 10.3390/jcm9113472 (PMC7692895; doi:10.3390/jcm9113472)
Supplement: Supplementary file 1 [file jcm-09-03472-s001.pdf]

Table S1. Univariate and multivariate analyses of baseline risk factors for death.

Risk factor	Unadjusted OR (95%CI)	*p*-value	Adjusted OR (95% CI)	*p*-value
**Age > 65 years old**	8.66 (5.26-15.16)	<0.001	**5.99 (3.42-11.05)**	**<0.001**
**High blood pressure**	2.93 (2.01-4.32)	<0.001	1.37 (0.79-2.38)	
**Diabetes mellitus**	1.57 (1.09-2.26)	0.013	0.92 (0.58-1.41)	
**Dyslipidemia**	1.82 (1.28-2.57)	<0.001	0.69 (0.44-1.09)	
**Tobacco consumption**	1.78 (1.19-2.65)	0.004	1.39 (0.87-2.20)	
**Active cancer**	3.74 (2.11-6.62)	<0.001	**2.87 (1.51-5.43)**	**0.001**
**Chronic kidney disease**	4.45 (2.92-6.79)	<0.001	**2.96 (1.79-4.89)**	**<0.001**
**Ischemic heart disease**	2.20 (1.37-3.49)	<0.001	0.83 (0.45-1.49)	
**Chronic heart failure**	3.63 (1.93-6.80)	<0.001	1.88 (0.89-3.98)	
**Previous antithrombotic drug**	3.50 (2.46-4.99)	<0.001	**1.67 (1.04-2.67)**	**0.033**
**Previous ACEIs/ARBs**	1.79 (1.27-2.53)	<0.001	0.97 (0.59-1.56)	
**Lymphopenia < 1000/µL**	2.74 (1.73-4.50)	<0.001	1.34 (0.89-2.05)	
**CRP ≥ 100 mg/ L (max)**	2.90 (1.93-4.48)	<0.001	1.31 (0.86-1.99)	
**D-dimer count (max) ≥ 1500 µg/L**	5.37 (2.53-13.2)	<0.001	1.43 (0.73-2.88)	

ACEI(s): Angiotensin–converting enzyme inhibitor(s); ARB(s): Angiotensin II receptor blocker(s); CI: Confidence interval; CRP: C-reactive protein; OR: Odds ratio; Bold: significant *p*-values

Table S2. Univariate and multivariate analyses of baseline risk factors for severe pneumonia (death, and/or OTI, and/or NIV, and/or HFNO, and/or oxygen flow of at least 5 L/min).

Risk factor	Unadjusted OR (95%CI)	p-value	Adjusted OR (95% CI)	*p*-value
**Age > 65 years old**	1.51 (1.12-2.02)	0.005	**1.78 (1.15-2.74)**	**0.009**
**Male sex**	2.21 (1.66-2.97)	<0.001	**1.58 (1.10-2.28)**	**0.012**
**High blood pressure**	1.71 (1.25-2.29)	<0.001	1.24 (0.75-2.04)	0.383
**Diabetes mellitus**	1.51 (1.09-2.09)	0.012	1.08 (0.69-1.67)	0.726
**Dyslipidemia**	1.26 (0.93-1.70)	0.13	0.82 (0.53-1.25)	0.416
**Obesity**	1.44 (1.05-2.00)	0.024	1.57 (1.02-2.39)	0.036
**Active cancer**	1.67 (0.93-3.09)	0.09	1.58 (0.79-3.26)	0.199
**Previous ACEIs/ARBs**	1.54 (1.14-2.09)	0.004	1.07 (0.66-1.72)	0.782
**Lymphopenia <1000/µL**	2.43 (1.75-3.39)	<0.001	1.45 (0.96-2.20)	0.07
**CRP ≥ 100 mg/L**	7.78 (5.58-10.97)	<0.001	**4.76 (3.27-6.97)**	**<0.001**
**D-dimer count ≥ 1500 µg/L**	8.94 (5.20-15.71)	<0.001	**4.24 (2.30-7.90)**	**<0.001**
**hs-cTnl ≥ 100ng/L**	3.12 (1.60-6.69)	0.001	1.73 (0.77-4.18)	0.306

ACEI(s): Angiotensin-converting enzyme inhibitor(s); ARB(s): Angiotensin II receptor blocker(s); CI: Confidence interval; CRP: C-reactive protein; OR: Odds ratio; hs-cTnl: High-sensitivity cardiac troponin.
